# A scoping review of mathematical models covering Alzheimer's disease progression

**DOI:** 10.3389/fninf.2024.1281656

**Published:** 2024-03-14

**Authors:** Seyedadel Moravveji, Nicolas Doyon, Javad Mashreghi, Simon Duchesne

**Affiliations:** ^1^Centre de recherche CERVO, Institut universitaire de santé mentale de Québec, Québec, QC, Canada; ^2^Département de mathématiques et de statistique, Québec, QC, Canada; ^3^Département de radiologie et médecine nucléaire, Université Laval, Québec, QC, Canada; ^4^Centre de recherche de l'Institut universitaire en cardiologie et pneumologie de Québec, Québec, QC, Canada

**Keywords:** amyloid beta, ordinary differential equations, partial differential equations, tau proteins, sensitivity analysis, mathematical model, validation, neurons

## Abstract

Alzheimer's disease is a complex, multi-factorial, and multi-parametric neurodegenerative etiology. Mathematical models can help understand such a complex problem by providing a way to explore and conceptualize principles, merging biological knowledge with experimental data into a model amenable to simulation and external validation, all without the need for extensive clinical trials. We performed a scoping review of mathematical models describing the onset and evolution of Alzheimer's disease as a result of biophysical factors following the PRISMA standard. Our search strategy applied to the PubMed database yielded 846 entries. After using our exclusion criteria, only 17 studies remained from which we extracted data, which focused on three aspects of mathematical modeling: how authors addressed continuous time (since even when the measurements are punctual, the biological processes underlying Alzheimer's disease evolve continuously), how models were solved, and how the high dimensionality and non-linearity of models were managed. Most articles modeled Alzheimer's disease at the cellular level, operating on a short time scale (e.g., minutes or hours), i.e., the micro view (12/17); the rest considered regional or brain-level processes with longer timescales (e.g., years or decades) (the macro view). Most papers were concerned primarily with amyloid beta (*n* = 8), few described both amyloid beta and tau proteins (*n* = 3), while some considered more than these two factors (*n* = 6). Models used partial differential equations (*n* = 3), ordinary differential equations (*n* = 7), and both partial differential equations and ordinary differential equations (*n* = 3). Some did not specify their mathematical formalism (*n* = 4). Sensitivity analyses were performed in only a small number of papers (4/17). Overall, we found that only two studies could be considered valid in terms of parameters and conclusions, and two more were partially valid. This puts the majority (*n* = 13) as being either invalid or with insufficient information to ascertain their status. This was the main finding of our paper, in that serious shortcomings make their results invalid or non-reproducible. These shortcomings come from insufficient methodological description, poor calibration, or the impossibility of experimentally validating or calibrating the model. Those shortcomings should be addressed by future authors to unlock the usefulness of mathematical models in Alzheimer's disease.

## 1 Introduction

### 1.1 Rationale

Alzheimer's disease (**AD**) is a neurodegenerative disorder that results in severely reduced cognition, loss of autonomy, eventual physical weakness, and death (McKhann et al., 2011). The occurrence of AD is strongly related to aging and the whole disease process covers a period estimated to last a minimum of 10–20 years before diagnosis (Kremer et al., 2011). This prodromal phase is thought to depend on physical, genetic, and epigenetic factors, all to be conclusively determined (Kremer et al., 2011).

Indeed, AD is difficult to diagnose in its earlier phases, since its etiology remains unclear. Multiple hypotheses have been proposed such as the beta-amyloid (*Aβ*) cascade hypothesis (Liu et al., 2015; Han and Shi, 2016; Kuznetsov and Kuznetsov, 2018a; Lindstrom et al., 2021); the tau protein hypothesis (Kuznetsov and Kuznetsov, 2018b); and the oligomer cascade hypothesis (Lindstrom et al., 2021). However, none has been proven beyond doubt nor has it led to a positive clinical outcome after interventions. Rather, it is becoming clear in the community that AD is a multi-factorial disease, influenced by several risk factors, and hence also multi-parametric. Therefore, early detection of AD and identification of a cure continue to be major challenges for the scientific community given both this lack of etiological clarity and apparent complexity.

A fundamental obstacle to AD research becomes the sheer number of biological, environmental, and psychosocial variables that must be considered, and by extension the geometrically increasing number of interactions between variables. These variables and interactions become difficult, if not impossible, to assess thoroughly via clinical trials, as the logistics of recruiting a sufficiently large number of individuals to achieve reasonable statistical power become daunting, without mentioning the decades-long timeframe required to follow these individuals along this neurodegenerative process. Conversely, while preclinical models can often provide clarity concerning specific variables and their interactions, they suffer from problems of generalizability to pseudo-sporadic AD in human populations.

Mathematical modeling on the other hand is a great tool to understand complex mechanisms such as AD. Mathematical models provide a way to explore and conceptualize principles by merging biological knowledge with experimental data into model simulations (Arkin and Schaffer, 2011). In mathematical models, physical reality is abstracted into entities and parameters that can help us more easily understand relationships. In the case of AD, mathematical models could help us figure out causal mechanisms and therefore propose targets for disease prevention. Accurate models should help with intervention planning and trial, by testing *in sillico* the potential effect of different drugs. Models however are only as good as the assumptions on which they are based. Indeed, if a model makes predictions that are out of line with observed results, or that cannot be verified experimentally altogether, either the entity relationships must be modified, or initial assumptions must be changed.

The development of personalized medicine is critical for any hope of clinical progress. This is exemplified in work such as that by Hao et al. (2022), in which the authors use mathematical modeling to determine the best intervention for any individual. From a mathematical point of view, this leads to interesting optimization problems.

### 1.2 Scope

The mathematical approaches used in models of AD are diverse, as there are different questions that several modeling formalisms aim to tackle. We focused our attention on models that considered AD progression, or a proxy of AD progression such as brain volume or the density of neural cells, as a state variable. The objective of such models is to relate causal factors such as the concentration of tau proteins and Abeta plaques to the death of neurons. This becomes immediately useful in a clinical context, as model outcomes can be related to biomarkers available using standard means (e.g., radiological imaging). Models in this family are often specified by systems of ODE or PED. Such models include Anastasio (2011), Anastasio (2011), Bertsch et al. (2017), Hao and Friedman (2016), Helal et al. (2014), Lindstrom et al. (2021), and Puri and Li (2010), which will be discussed in more detail in the rest of this review.

### 1.3 Objectives

To guide our group in the elaboration of a comprehensive, multi-factorial mathematical model of AD, we elected to perform a scoping review of this nascent literature, the results of which are presented in the following sections. We paid particular attention to three aspects of mathematical modeling: first, how authors addressed continuous time, as relevant in describing the evolution of biological processes involved in AD; secondly, how models were solved numerically, given these different time scales at which the interactions between variables operate; and finally, how were high dimensionality and non-linearity of models managed, up to and including parameter sensitivity.

## 2 Methods

### 2.1 Eligibility criteria and information sources

We performed a scoping review based on the PRISMA standards (Page et al., 2021). We searched the PubMed database for peer-reviewed, original research journal papers in English published through March 29, 2022. Our search terms were “Mathematical model,” “Alzheimer's disease,” and “Not an animal.” In our search strategy, the use of only “Alzheimer's disease” but not terms such as “tauopathy” or protein “misfolding” was guided by the goal to be not only sensitive to AD but specific. As for the choice of “Mathematical model,” other search terms such as “Computational model” would be reasonable but did not yield significant advantages.

### 2.2 Search strategy and selection process

Using the Covidence systematic review system (Melbourne, Australia), we first removed duplicate entries after the initial search and then screened articles based on titles and abstracts. We then completed a full-text evaluation of each article. Our goal was to identify multi-factorial mathematical models of AD applied to a human population. Consequently, we disregarded approaches that were too narrow (e.g., only one protein) or animal-centered.

### 2.3 Data collection process and data items

We extracted the following characteristics from all included studies (cf. Table 1): Title, Lead author contact details, Country(ies) of origin of authors, Aim of study, Temporal scale (micro or macro) (see Section 3.2), Multi-structure or not (see Section 3.2), Validated or not, Interesting summary figure, Study funding sources, Possible conflicts of interest for study authors. Information regarding mathematical modeling was also extracted (cf. Table 2), including which mathematical formalism was used. If the model relied on ODE, what solver was used? Given the solver, was it adapted to stiff equations? We also extracted information on the concepts and entities that were captured by each model.

**Table 1:** Main characteristics of the reviewed papers.

	**References**	**Title**	**Object**	**Time scale**
1	Anastasio (2011)	Data-driven modeling of Alzheimer's disease pathogenesis.	Aβ	Micro
2	Bertsch et al. (2017)	Alzheimer's disease: a mathematical model for onset and progression.	Aβ	Macro
3	Fornari et al. (2019)	Prion-like spreading of Alzheimer's disease within the brain's connectome.	Tau protein	Micro
4	Han et al. (2020)	Computational modeling of the effects of autophagy on amyloid-β peptide levels.	Aβ	Micro
5	Han and Shi (2016)	A Theoretical Analysis of the Synergy of Amyloid and Tau in Alzheimer's Disease.	Aβ, Tau	Micro
6	Hao and Friedman (2016)	Mathematical model on Alzheimer's disease.	Aβ, Tau, Astrocytes, Microglia, Macrophages, GSK3, NFT, APP, TNF_α_, IL-10, TGF, MCP-1, ROS, HMGB1	Macro
7	Helal et al. (2014)	Alzheimer's disease: analysis of a mathematical model incorporating the role of prions.	Aβ oligomers, Aβ plaques, and cellular prion proteins	Micro
8	Helal et al. (2019)	Stability analysis of a steady state of a model describing Alzheimer's disease and interactions with prion proteins.	Aβ peptides, Aβ oligomer	Micro
9	Hoore et al. (2020)	Mathematical Model Shows HowSleep May Affect Amyloid-β Fibrillization.	Aβ, Aβ oligomer, microglia, CSF	Macro
10	Kuznetsov and Kuznetsov (2018a)	How the formation of amyloid plaques and neurofibrillary tangles may be related: a mathematical modeling study.	Tau„ APP external Aβ plaques, intracellular tangles	Micro
11	Kuznetsov and Kuznetsov (2018b)	Simulating the effect of the formation of amyloid plaques on the aggregation of tau protein.	Aβ, Tau, APP, neurofibrillary tangles	Micro
12	Lindstrom et al. (2021)	From reaction kinetics to dementia: A simple dimer model of Alzheimer's disease etiology.	Aβ Oligomer, Aβ monomers, dimers, trimers	Macro
13	Liu et al. (2015)	Evaluating Alzheimer's Disease Progression by Modeling Crosstalk Network Disruption.	Aβ, Aβ Oligomer, P-Tau, Tau, MCI, GSK3	Micro
14	Pallitto and Murphy (2001)	A mathematical model of the kinetics of beta-amyloid fibril growth from the denatured state.	Aβ monomer to oligomer	Micro
15	Petrella et al. (2019)	Computational Causal Modeling of the Dynamic Biomarker Cascade in Alzheimer's Disease.	Aβ, tau, tau pathology (p-tau)	Micro
16	Proctor and Gray (2012)	A unifying hypothesis for familial and sporadic Alzheimer's disease.	Aβ, Tau	Macro
17	Puri and Li (2010)	Mathematical modeling for the pathogenesis of Alzheimer's disease.	Aβ, microglia, astroglia	Macro

**Table 2:** A summary of mathematical approaches, solvers, and sensitivity analysis of reviewed papers.

	**References**	**Model type**	**If ODE, what solver was used**	**Sensitivity Analysis**
1	Anastasio (2011)	Not mentioned	Maude	No
2	Bertsch et al. (2017)	PDE	Not mentioned	No
3	Fornari et al. (2019)	PDE, ODE and Graph	Not mentioned	No
4	Han et al. (2020)	ODE	5th order Runge-Kutta method	No
5	Han and Shi (2016)	Seven ordinary equations	Not mentioned	No
6	Hao and Friedman (2016)	ODE and PDE	Not mentioned	Yes
7	Helal et al. (2014)	ODE	Not mentioned	No
8	Helal et al. (2019)	ODE	Not mentioned	No
9	Hoore et al. (2020)	ODE	Not mentioned	Yes
10	Kuznetsov and Kuznetsov (2018a)	PDE	MATLAB's BVP4C solver	No
11	Kuznetsov and Kuznetsov (2018b)	PDE	Not mentioned	No
12	Lindstrom et al. (2021)	ODE and PDE	Python	Yes
13	Liu et al. (2015)	Not mentioned	Not mentioned	No
14	Pallitto and Murphy (2001)	ODE	Not mentioned	No
15	Petrella et al. (2019)	ODE	MATLAB	No
16	Proctor and Gray (2012)	Not mentioned	Not mentioned	No
17	Puri and Li (2010)	ODE	Not mentioned	Yes

### 2.4 Study risk of bias assessment

Two impartial reviewers independently conducted this process (S.M. and S.D.), and consensus solved conflicts.

### 2.5 Effect measures

Providing information on specific outcomes or effect measures does not apply in the context of this scoping review.

### 2.6 Synthesis methods

The studies were grouped according to the mathematical formalism used in the model. Concerning heterogeneity and sensitivity, we paid attention to the validation approaches used in the investigated papers.

### 2.7 Reporting bias assessment

As the analysis was done by impartial and independent reviewers, there was no reporting bias.

### 2.8 Certainty assessment

We provide a narrative synthesis of the available literature, highlighting the strengths and weaknesses of the included studies and identifying areas for future research in Section 3.

## 3 Results

### 3.1 Study selection

**Figure 1:**
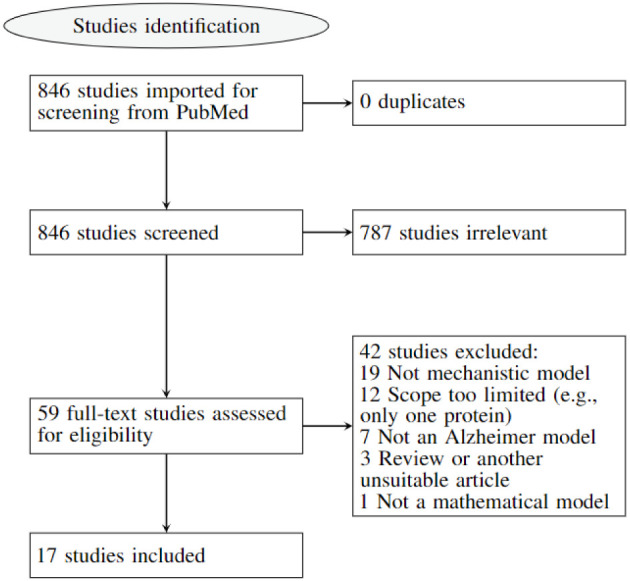
PRISMA flow chart diagram. This diagram illustrates how the papers were selected for the scoping review and why some were excluded.

After our search, 846 studies were uploaded from PubMed to Covidence on March 29, 2022. Two reviewers independently screened the titles and abstracts to exclude papers not relevant to this review. If they disagreed on a paper, they re-evaluated it together and reached a consensus. After removing duplicates and reviewing titles and abstracts, 59 references appeared to meet our criteria. After full-text review, 41 were further excluded due to them not proposing a mechanistic model (*n* = 19); having a limited scope (e.g., describing only one protein) (*n* = 12); not being related to Alzheimer's disease (*n* = 7); or being a review or another unsuitable article form (*n* = 4) (see Figure 1), leaving 17 papers to be studied.

We aimed to select studies that relate the onset and temporal evolution of AD to neural death and underlying biological mechanisms and assess validity in the context of clinical (human) care. Even if they were less represented, we wanted to investigate papers with multifactorial models as we believe they were more likely to provide useful insights regarding disease processes. We wanted to exclude purely statistical studies or studies that did not propose a cause for AD, hence the reference to mechanistic models.

### 3.2 Study characteristics

The 17 papers were published between 2001 and 2021. The primary authors' countries of origin were the United States (*n* = 11) and others (Algeria, China, France, Germany, Italy, and the United Kingdom).

The majority of articles modeled AD at the cellular level, operating on a short time scale (e.g., minutes or hours), i.e., the *micro* view (12/17), while the rest of the models considered regional or brain-level processes with longer timescales (e.g., years or decades) (the *macro* view). Most of the papers were concerned primarily with *Aβ* (*n* = 8); a few modeled both *Aβ* and tau proteins (*n* = 3); and some considered more than these two factors (*n* = 6) (see Table 1).

### 3.3 Mathematical approaches

Mathematical approaches are summarized in Table 2. Various approaches were used, such as partial differential equations (PDE; *n* = 3), ordinary differential equations (ODE; *n* = 7), and both PDE and ODE (*n* = 3), while for the remainder (*n* = 4), the type of model was not presented and only results were discussed. Of these last articles, one study used *Maude*, a language able to represent and evaluate models created using equations and rules (Anastasio, 2011). The choice of using either ODEs or PDEs depended on how the spatial propagation of AD in the brain was considered. Models relying on PDEs treat the brain as a continuous medium with realistic geometry, while models using ODEs either neglect the spatial component altogether or consider only a few homogenized regions. We give more details in Section 4.4.

### 3.4 Risk of bias in studies

The risks of bias are summarized in Table 3.

**Table 3:** Evaluation of the internal and external validity of the reviewed papers.

	**References**	**Given equations and initial conditions**	**Given parameters and their units**	**Sensitivity Analysis**	**Given code**	**Information on model solver**	**Internal validity degree**	**external validity**
1	Anastasio (2011)	No	No	No	No	Maude	1/5	No
2	Bertsch et al. (2017)	Yes	No	No	No	No	1/5	No
3	Fornari et al. (2019)	Yes	No	No	No	No	1/5	No
4	Han et al. (2020)	Yes	Yes	No	No	5th order Runge-Kutta method	3/5	No
5	Han and Shi (2016)	Yes	No	No	No	No	1/5	No
6	Hao and Friedman (2016)	Yes	Yes	Yes	No	No	3/5	No
7	Helal et al. (2014)	Yes	No	No	No	No	1/5	No
8	Helal et al. (2019)	Yes	Yes	Yes	No	No	3/5	No
9	Hoore et al. (2020)	Yes	Yes	No	No	No	2/5	Yes
10	Kuznetsov and Kuznetsov (2018a)	Yes	No	No	No	MATLAB's BVP4C solver	2/5	No
11	Kuznetsov and Kuznetsov (2018b)	Yes	No	No	No	No	1/5	No
12	Lindstrom et al. (2021)	Yes	Yes	Yes	Yes	Python	5/5	Yes
13	Liu et al. (2015)	No	No	No	No	No	0/5	Yes
14	Pallitto and Murphy (2001)	Yes	Yes	No	No	No	2/5	Yes
15	Petrella et al. (2019)	Yes	Yes	No	No	MATLAB	3/5	No
16	Proctor and Gray (2012)	available online	available online	No	No	No	2/5	No
17	Puri and Li (2010)	Yes	Yes	Yes	No	No	3/5	No

### 3.5 Results of individual studies

The results of individual studies are summarized in Table 1.

### 3.6 Results of syntheses

Of the ODE models, four had components evolving over several time scales, from cellular reactions lasting minutes to disease development over the years; the other six had a single time scale. The description of phenomena with characteristic time constants over several orders of magnitudes leads to so-called *s*tiff systems of ODEs. The resolution of stiff differential equations poses several numerical difficulties, and the choice of an inappropriate resolution method can lead to false conclusions. In general, we found that there was not enough support or information given in the articles about which solvers and numerical tools were used. Only four papers (Puri and Li, 2010; Hao and Friedman, 2016; Fornari et al., 2019; Lindstrom et al., 2021) out of 10 mentioned which environment they used to solve their ODE system (three used MATLAB and one used Python Lindstrom et al., 2021). Among these four papers, only two mentioned which numerical solvers were used. This limits our appreciation of their work.

Performing sensitivity analysis (**SA**) in mathematical models is critical to identify which parameters have an important impact on the solutions and to quantify uncertainty. SA can identify crucial model inputs (parameters and initial conditions) and quantify the impact of input uncertainty on the model's output(s) (Marino et al., 2008). We thus investigated whether the reviewed papers performed SA and, if so, which approach was used. We found that little attention has been given to this crucial topic since SA is covered, but only for a small number of papers (4/17).

### 3.7 Reporting biases and certainty of evidence

As we relied only on the PubMed database and limited ourselves to a specific set of keywords for our search, we may have missed relevant studies.

## 4 Discussion

### 4.1 Summary of findings

The negative impact of AD on patients, caregivers, and society is well established. Given that there is no known cure for this disease, identifying relationships between biological factors leading to its causality is paramount, as any insight can inspire therapeutic approaches to delay or stop its progression, reducing suffering and costs (Alzheimer Society of Canada, 2022). Mathematical models can be extremely useful in the quest to decipher the interactions between different parts of a complex system and to understand how acting on one element of the system will impact global outcomes. In this scoping review, we identified and presented findings from 17 papers with mathematical models of AD.

### 4.2 Proposed mechanisms

Most of the investigated papers considered either the amyloid beta or the tau proteins (or a combination of both) as the underlying mechanism causing the onset of AD. It is proposed that these pathways will cause neural death. It would be interesting to investigate other mechanistic hypotheses.

### 4.3 Mathematical model families

**Time-continuous longitudinal models of AD onset and progression**. As mentioned previously, we focused our scoping review on models that considered AD progression, or a proxy of AD progression, as a state variable. Other reviews have discussed the mathematical modeling of different elements involved in AD progression, such as amyloid beta aggregation and tau proteins (Vosoughi et al., 2020), the impact of interregional connectivity (Torok et al., 2023), and protein misfolding (Carbonell et al., 2018). Our approach was different, as we aimed to focus on assessing the validity of mathematical models of AD, including their adequacy with clinical results. This validity is usually taken for granted and we think it is worthwhile to question it.

**Models of spatial propagation and fractional derivative models**. Some models focus on the description of the spatial propagation of AD or its underlying factors, such as Aβ plaques or tau proteins. These models can rely on partial differential equations (PDEs) or network theory (Bertsch et al., 2017; Fornari et al., 2019). From a mathematical point of view, diffusion in general and diffusion in the brain in particular can be described by fractional derivatives. The mathematical tool of fractional derivatives is well suited, for instance, to describe Brownian motion. In Alkahtani and Alzaid (2021), the authors claim that as the spatial propagation of the disease is not well understood, it is worth using a model that accounts for non-localities. To achieve this, they use the mathematical formalism of a Caputo derivative. In Angstmann et al. (2016), the authors use Riemann-Liouville fractional derivatives to describe the spread of pathogenic proteins. In Mohammad and Trounev (2021), the authors develop a fractional order PDE model to describe the evolution of AD. Their paper emphasizes the development of the model. In Pawar and Pardasani (2023), the authors use fractional calculus to describe calcium dynamics in cells and its impact on amyloid beta. Finally, in Karaoulanis et al. (2023), the authors use fractional derivatives to describe anomalous diffusion in brain tissue.

**Models of Amyloid beta aggregation** From a mathematical perspective, describing the aggregation of amyloid beta from monomers to plaques poses many difficulties. A challenge comes from the fact that chains of arbitrary length can merge or dissociate, leading to a high-dimensional problem. A common approach is to group the chains according to their length, considering, for instance, monomers, oligomers, and plaques (Hao and Friedman, 2016). Another mathematical approach is to use the Smoluchowski equations (Bertsch et al., 2017).

**Models describing the impact of AD on neural network performance** Some mathematical models focus not on the continuous temporal evolution of AD (or of a proxy of AD) but rather on the effect of AD on brain behavior. There is a very rich literature on mathematical models of biological neural networks, which are often represented as dynamical systems of high dimensions. AD or other neurodegenerative diseases can be represented in these models by a loss of neurons or a loss of connectivity between neurons. It is interesting to see how such a loss can lead to a loss of performance for the system. Such investigations can shed light on the precise relationship between loss of neurons, loss of synapses, impaired synaptic activity, and impaired neural activity on cognitive functions. Interestingly, some topological properties of neural networks, such as the existence and nature of dynamical attractors, can be altered by proxies in AD. In Karageorgiou and Vossel (2017), the authors investigate the dynamical attractor of brain rhythm and show that it can be disrupted by AD. In Alderson et al. (2018), the authors consider the brain connectome. They consider lesions in the connectome as a result of AD which leads to a loss of neurons and connections. They investigate how such lesions affect the dynamic behavior of the system. The hippocampus plays a paramount role in memory, and how its behavior is affected by AD is of critical interest. In Kanagamani et al. (2023), the authors build a network model of hippocampal memory and investigate how it is affected by AD-like conditions, while Jiang et al. (2020), emphasize the investigation of cholinergic action. Recurrent networks are an important family of artificial neural networks with feedback and may more accurately mimic biological networks. In Bachmann et al. (2013), the authors investigate how AD might affect the computations made by such networks.

### 4.4 Mathematical considerations

Mathematical models using continuous time, as is relevant in the case of AD, can rely either on systems of ODEs when the description of brain geometry is omitted or greatly simplified or on systems of PDEs when a continuous spatial description of the brain is used. Either approach is *a priori* possible, depending on whether one wants to emphasize or not the spatial nature of the propagation of the disease.

Models described by a system of ODEs can be written as


$$\begin{array}{l} \frac{d\vec{y}}{dt}=\vec{f}\left(\vec{y},\vec{\theta },t\right) \end{array}$$

where arrows denote vector quantities, $\vec{y}$ describes the state of the system, and $\vec{\theta }$ are the parameters that are usually either found in the literature or determined by fitting the simulation results to experimental data.

In models described by PDEs, the left-hand side becomes $\frac{\partial y}{\partial t}$, and the right-hand side now depends on the spatial derivatives of *y* and usually involves diffusion or transport terms.

In either case, systems of equations describing the evolution of AD involve a large number of parameters describing biophysical quantities such as the rate of neural death or the rate of *Aβ* aggregation. Determining the value of these parameters is challenging. Given the various components involved in AD onset and progression, mathematical models will contain several variables. Also, as is often the case with biological systems, models describing the progression of AD tend to be non-linear. The interaction between each pair of variables will need to be quantified by a distinct parameter capturing its relative importance. Since many or most of these parameters cannot be directly measured, the objective determination of their values (or model calibration) is challenging. The process of calibrating a mathematical model should ideally be done by comparing the model's outputs to actual clinical, epidemiological, or experimental data (Alarid-Escudero et al., 2018). Then, parameters should be chosen to minimize the difference between the model predictions and the observed data. The possibility of fully characterizing a model is dependent on the quantity and quality of available data (Thacker et al., 2004). In the case of AD, recent initiatives aimed at the acquisition of large publicly accessible datasets promise to increase the relevance and importance of mathematical models.

We noticed that in papers analyzed for this review, there was a wide discrepancy between the approaches taken to determine and report model parameters. Some authors used parameter values found in other modeling or experimental studies or derived them from theoretical calculations (e.g., Hao and Friedman, 2016; Hoore et al., 2020; Lindstrom et al., 2021). However, some papers did not explain either the provenance of their parameters or their calibration process (e.g., Anastasio, 2011; Han and Shi, 2016; Bertsch et al., 2017; Kuznetsov and Kuznetsov, 2018a,b; Fornari et al., 2019). Others listed parameter values without providing references or calculations to justify them (e.g., Helal et al., 2014; Petrella et al., 2019). Only a few papers provided experimental results to support their choice of parameter values (e.g., Pallitto and Murphy, 2001; Puri and Li, 2010; Liu et al., 2015). Studies should specify their methodology for parameter calibration and model validation to increase our ability to judge whether the model reached accurate predictions (Qiu et al., 2018). When it is impossible to assess model parameters directly, their values should be determined by comparing the model's output with experimental or clinical observations. In this case, optimization techniques are needed to minimize the difference between model predictions and observations (Qiu et al., 2018). Details of these techniques (and ideally the code to perform them) should also be provided, as optimization in highly dimensional non-linear systems is far from a trivial task (Qiu et al., 2018). Depending on data type and data quality, there might be a great deal of uncertainty concerning parameter values inferred in this way, hence the importance of specifying methodological details to increase reproducibility (Qiu et al., 2018).

A second mathematical challenge comes from the fact that time scales ranging over several orders of magnitudes are typically involved in biomathematical models describing the onset and progression of AD. For instance, such models could describe rapid protein transformation as well as the progression of the disease over several years. Lindstrom et al. (2021) wrote: “There are fast time scales for dimer dissociation (*ms*); intermediate time scales for monomer decay (*h*); and longtime scales for changes in kinetic rate constants and loss of neuronal health (*decades*).” The work by Puri and Li (2010) is also a good example of this, with parameters scaling from 10^−5^ to 1 (1/*year*). From a mathematical standpoint, systems with components behaving over several time scales are described by stiff differential equations (Nasarudin et al., 2020) for which numerical resolution is challenging and necessitates well-adapted specific approaches.

When modeling phenomena occurring on different time scales (say, we both consider a fast and a slow process), one must use computational time steps small enough to capture the fast process but run the model long enough to describe the slow process. This leads to a high computational cost and raises numerical challenges (Kreiss, 1979; Burden and Faires, 1993). Other approaches, such as fast-slow analysis, can be used to resolve systems with different time scales, though they come with their challenges (Bertram and Rubin, 2017).

We found in our review that many authors did not detail which solver they used and/or did not give sufficient information on the numerical methods they used to solve their system of equations. To wit, there are multiple stiff ODE solvers in MATLAB (e.g., ode15s, ode23s, ode23t, and ode23tb) or Python (BDF and Radau in the SciPy library). This makes it unclear whether the numerical difficulties associated with the stiffness of the model were appropriately handled. Unfortunately, authors missed including this vital aspect of their research in the description of their work, as it is necessary for reproducibility.

Finally, a third challenge in the development and analysis of high-dimension mathematical models with a large number of parameters is related to sensitivity analysis and uncertainty quantification. Performing sensitivity analysis is essential to assess the relative contribution of each parameter (Helal et al., 2019). Parameters having little impact on the model outcome cannot be determined only by comparing model predictions to actual data (Qiu et al., 2018). Several mathematical approaches can be used to perform sensitivity analysis, each providing complementary information (Marino et al., 2008; Lindstrom et al., 2021). In Hao and Friedman (2016), the authors performed a sensitivity analysis using Latin hypercube sampling to generate 2, 000 samples. In each sample, the parameter values were chosen randomly between one-half and twice their normal values. The authors calculated partial rank correlation coefficients and *p*−values for the density of neurons and the concentration of astrocytes at time *t* = 10 years. Another approach used by Lindstrom et al. (2021) was to evaluate how tiny perturbations (1% change in a parameter value) affected the model outcome and then evaluate the probability that the AD development rate differed significantly from their initial estimate. Puri and Li (2010) took into consideration the sensitivity of the outcome after 20 years to changes in parameter values. They used a 62.5% perturbation range for each parameter value. They reported strong, moderate, or weak effects for each parameter, along with a short explanation of their sensitivity analysis process (Puri and Li, 2010). Unfortunately, not all authors were equally forthcoming. Some (e.g., Helal et al., 2019) claim to have performed a sensitivity analysis but did not report their method. Most others simply did not perform such an analysis. We propose as a good practice that papers describing a mathematical model of AD should include a sensitivity analysis, complete with a description of the approach, code or pseudo-code, and numerical estimates.

Anastasio (2011) used the MAUDE software to check the impact of various components, such as genes, on AD pathogenesis. A few mathematical details were provided in the paper. Bertsch et al. (2017) solved the Smoluchowski coagulation equation to describe the aggregation and diffusion of amyloid beta. This led to a PDE system that was discretized on a uniform grid. Fornari et al. (2019) was quite rich from a mathematical perspective. The authors solved a system of PDEs and also used network theory. A statistical analysis was performed on the results of their models. In Han et al. (2020), the author solved a system of ODES. Their model assumed a four-compartment description of the autophagy process. The authors obtained a rather simple system of eight equations. Han and Shi (2016) did not rely on a dynamical model per se. The authors used different simple equations to test whether there is a synergy between tau proteins and amyloid beta in AD. In Hao and Friedman (2016), the authors used an ODE system to predict the loss of neural density during aging, which is used as a proxy for AD progression. The model contained 16 state variables and several tens of parameters. The authors performed sensitivity analysis to identify the relative importance of several parameters. They investigated the synergy between different *in silico* treatment approaches. Helal et al. (2014) solved an equation system combining partial differential equations and integro differential equations. In their model, the temporal evolution of the concentration of soluble amyloid beta depended on the spatial average of plaque concentration.

In Helal et al. (2019), the authors used a high-dimensional system of ODEs to describe amyloid beta aggregation. They treated the quantity of oligomers of each possible size as a state variable. It is noteworthy that the authors provided rigorous proof of the existence of solutions. In Hoore et al. (2020), the authors used a simple 2-dimensional ODE model, which requires 11 scalar parameters to be fully specified. The authors performed an extensive numerical analysis of the model. In Kuznetsov and Kuznetsov (2018a) and Kuznetsov and Kuznetsov (2018b), the authors described the transport of APP and tau proteins along axons. Their model is a rather complex system of partial differential equations. In contrast to other models discussed in this review, the authors considered a one-dimensional geometry corresponding to a wire-like axon. Lindstrom et al. (2021) considered both a system of ODEs and a system of PDEs. They described the concentration of amyloid beta monomers and dimers as well as cell viability. Their model has three state variables and 20 scalar parameters. Parameter estimation was described in detail. Liu et al. (2015) performed Monte Carlo simulations on a network describing the progression of AD. They performed several random perturbations on the network and evaluated the probability that these perturbations disrupted the model outcome. Pallitto and Murphy (2001) used a high-dimensional system of ODEs to describe the growth of amyloid fibrils. Their model was based on biophysical principles. Petrella et al. (2019) used a simple model of five ordinary differential equations and about 30 parameters relating cognitive decline to the concentration of amyloid beta and tau proteins. This model was closely related to that of Hao and Friedman (2016). In Proctor and Gray (2012), the authors implemented a dynamic stochastic model in the *Systems Biology Markup Language*. A few details were provided concerning the mathematical implementation. They used their model to compare different hypotheses related to the interactions between tau proteins and amyloid beta. Finally, in Puri and Li (2010), the authors investigated an ODE system with seven state variables and 17 scalar parameters. Their relatively simple model described the impact of amyloid beta on neural death.

It is to be noted that given the complexity of the resulting systems of equations, analytic approaches aiming to provide close solutions were not used in the reviewed papers except (Lindstrom et al., 2021). While it could be interesting to perform asymptotic or approximation analysis in the models that were used in the reviewed papers, we found that numerical treatment and numerical resolution were by far the most prominent approaches.

As a matter of course, AD models eventually need to be set within the spatial topography of the brain, with considerations of inter-structure connectivity and local/regional influence on initial conditions and parameters. PDEs can be used for this purpose, taking the geometry of the brain into account, and therefore play a prominent role in the mathematical description of the disease's onset and progression. However, it seems that there is some debate in the literature about whether amyloid and tau pathology in Alzheimer's disease is mainly driven by emergence or spread. Emergence refers to the idea that pathological entities form in different brain regions due to local factors, such as age, genetics, inflammation, or synaptic activity. Spread refers to the idea that pathological entities propagate from one region to another through direct or indirect mechanisms, such as transneuronal transmission, extracellular diffusion, or vascular transport. One way to model the emergence of amyloid and tau pathology is to assume that the aggregation and removal rates of these proteins depend on local variables. For example, Fornari et al. (2019), considered the effect of synaptic activity and neuronal damage on the model parameters, and Hao and Friedman (2016), considered the effect of cytokines produced by dead neurons. They obtained a system of PDEs that was discretized on a realistic geometry of the human brain, obtained from magnetic resonance imaging (MRI) data. They also used network theory to analyze the connectivity and vulnerability of different brain regions to the disease. They found that their model can reproduce the observed patterns of *Aβ* and tau accumulation and spreading in AD, as well as the regional differences in disease susceptibility and progression. Another way to model the spread of amyloid and tau pathology is to assume that these proteins diffuse through the brain tissue or along neural fibers. For example, Lindstrom et al. (2021) proposed a simple dimer model of Alzheimer's disease etiology that links *Aβ* assembly to oligomer-induced neuronal degeneration. They used a combination of ODEs and PDEs to describe the production, aggregation, diffusion, and elimination of amyloid dimers. They used their model to explain various aspects of AD, such as the rapid growth of disease incidence with age, the clinical progression in genetic forms of AD, the changes in hippocampal volume, the AD risk after traumatic brain injury, and the spatial spreading of the disease. Bertsch et al. (2017) used a reaction-diffusion equation of the form


$$\frac{\partial u}{\partial t}=D\nabla^{2}u+f\left(u\right)$$

where *u* is the concentration, *D* is the diffusion coefficient, and ∇^2^ is the Laplacian operator. The function *f*(*u*) can, for example, represent the aggregation and removal of amyloid beta. In Bertsch et al. (2017), the function *f* corresponds to the precise but complicated Smoluchowski coagulation equation, which describes every possible size of the amyloid beta chain by a different state variable. In Alkahtani and Alzaid (2021), the authors replace conventional PDEs with a version involving fractional derivatives which they claim leads to a more realistic model. These papers did not explicitly model the emergence of amyloid and tau pathology, but they did account for the age-dependent changes in the model parameters. Therefore, different papers had different ideas in mind when using PDEs to model Alzheimer's disease. Some focused on the emergence of pathology, while others focused on the spread of pathology. However, there may be a bit of both in reality, and the relative contribution of each factor may vary depending on the stage and subtype of the disease.

### 4.5 Internal and external validity

Determining internal and external validity is a critical but sometimes overlooked aspect of mathematical modeling. Table 3 summarizes the extent to which papers satisfied the criteria of internal and external validity.

Internal validity is related to model verification and takes into account the mathematical underpinning of the model. An important aspect of internal validity is the verification of the accuracy of numerical solutions. For a mathematical model to be considered internally valid, the software and numerical methods utilized in solving the model should be specified. The code or pseudocode used should be accessible and subject to verification. Techniques like static and dynamic testing should also be used to confirm the reliability, efficiency, and robustness of the code (Thacker et al., 2004). To determine the extent of the internal validity of the papers, we checked whether the authors provided explicit equations, initial conditions, and parameter values, and if so, whether this was sufficiently justified. We also verified if codes or pseudo-codes were provided and if sensitivity analyses were performed.

External validity, also known as model validation, compares simulation results and model predictions to experimental data, which is used to confirm that the predictions are accurate (Thacker et al., 2004). As can be appraised from Table 3, few articles were considered both internally and externally valid. Future reports are encouraged to provide information regarding validity as a general condition for model acceptability.

### 4.6 Limitations

As a scoping review, we used a limited set of search terms (without synonyms) and only one database (PubMed). This means that many studies will have been missed. Notable among the studies that were not detected by our initial search are Weickenmeier et al. (2019), Thompson et al. (2020) both providing interesting mechanistic models of neurodegeneration.

However, commonalities emerged from the representative sample of studies that were reviewed. A second limitation comes from the wide scope of modeling that was proposed by the authors. While many looked at individual steps in a pathological cascade (especially that related to amyloid accumulation) few modeled the entire disease course from onset to neuronal death.

## 5 Conclusion

Since there is no effective treatment for AD, efforts to understand its etiology are essential as a first step to devising any intervention aimed at delaying its onset. More research is therefore needed in this direction. Computational models can help to disentangle the roles of the various elements involved in the onset and development of AD. That said, close attention must be paid to various aspects of these models for them to be valid and useful. For instance, the choice of an appropriate numerical solver is essential to avoid irrelevant results, while a detailed description of the numerical approaches used to solve the problem is necessary to make the results reproducible. Providing code and equations is also a good practice. Using biologically realistic parameters in mathematical models of AD is essential, as badly calibrated models can yield irrelevant predictions, and details of how these parameters are chosen should be given. As models describing AD tend to be higher-dimensional, to be described by a large number of parameters, and to be non-linear, sensitivity analysis is required to identify which parameters play a critical role and which ones are less important. Systematically fulfilling these requirements would improve our confidence in the models being proposed. Hopefully, this will help research reach a stage where mathematical models of AD can make significant, testable predictions and play a significant role in the development of new therapeutic approaches.

## Author contributions

SM: Writing – review & editing, Writing – original draft, Software, Methodology, Investigation, Data curation. ND: Writing – review & editing, Supervision, Conceptualization. JM: Writing – review & editing, Supervision. SD: Writing – review & editing, Validation, Supervision, Resources, Funding acquisition, Conceptualization.
